# Hypercalcemia and Osteolytic Lesions as Presenting Symptoms of Acute Lymphoblastic Leukemia in Children: Case Report and Literature Review

**DOI:** 10.3389/fped.2022.923297

**Published:** 2022-06-09

**Authors:** Min Chen, Jiaqi Ni, Xiaoxi Lu

**Affiliations:** ^1^Department of Pharmacy, West China Second University Hospital, Sichuan University, Chengdu, China; ^2^Evidence-Based Pharmacy Center, West China Second University Hospital, Sichuan University, Chengdu, China; ^3^Key Laboratory of Birth Defects and Related Diseases of Women and Children, Sichuan University, Ministry of Education, Chengdu, China; ^4^West China School of Pharmacy, Sichuan University, Chengdu, China; ^5^Department of Pediatric Hematology and Oncology, West China Second University Hospital, Sichuan University, Chengdu, China

**Keywords:** acute lymphoblastic leukemia, children, hypercalcemia, calcitonin, pamidronate

## Abstract

Acute lymphoblastic leukemia (ALL) presenting with hypercalcemia and osteolytic lesions is rare and unusual in childhood. We report a case of a 13-year-old boy with ALL who presented with intermittent fever, nausea, vomiting, and increasing lower limb pain. Skeletal X-rays and CT scan showed severe osteolytic lesions of the skull and extremities. Physical examination revealed multiple inguinal lymph nodes. Laboratory tests demonstrated severe hypercalcemia (Ca > 3.49 mmol/L), decreased parathyroid hormone (PTH), and vitamin D level. Despite a normal complete blood count and the absence of circulating blasts, bone marrow biopsy revealed B-precursor ALL. Hypercalcemia was initially treated with intravenous isotonic sodium chloride and furosemide but the serum calcium level was not normalized. It was successfully managed with calcitonin and pamidronate afterward. Later, the child responded well to chemotherapy and continued with consolidation treatment. The clinical condition was stable, and the bone marrow revealed complete remission. This case indicated that hypercalcemia alone or combined with osteolytic lesions can be the only presenting symptom of ALL in children. Diagnostic errors may occur especially when combined with the absence of circulating blasts in the peripheral blood smear. Bone marrow aspiration should be considered to confirm the diagnosis.

## Introduction

Acute leukemia (AL) is the most common malignant neoplasm in children and adolescents. Acute lymphoblastic leukemia (ALL) accounts for around 75% of the cases of AL in childhood (1). ALL usually presents with fever, pale appearance, bleeding, joint pain, lymphadenopathy, and hepatosplenomegaly. Hypercalcemia combined with osteolytic lesions is very rare in children (2–4). This article reports the clinical characteristics, pathogenesis, treatment, and prognosis of a child with ALL who presented with hypercalcemia and osteolytic lesions as main symptoms.

## Research Methods

### Case Analysis

A case presented with recurrent left lower limb and hip pain, combined with intermittent fever, nausea, and vomiting was reported. The main clinical presentations, physical examination and laboratory results, and the treatment outcome were analyzed retrospectively.

### Literature Review

PubMed and one Chinese database (CNKI) were searched with keywords including acute lymphoblastic leukemia and hypercalcemia. Literature in Chinese and English language on children (≤ 18 years old) with ALL complicated with hypercalcemia was retrieved. Descriptive analysis was conducted to reveal the clinical characteristics and treatment options for the disease.

## Results

### Case Report

#### Case Presenting

A 13-year-old boy presented to us with left lower limb pain for over 2 months, recurrent vomiting for 20 and 40 days after left femoral neck fracture operation on 5 May 2020.

Initially, the disease was characterized by repeated pain with progressive aggravation in the left lower limb and hip, limited body movement, claudication without paresthesia, limb numbness, morning stiffness, or rash. The case was complicated with intermittent fever and epistaxis, without pallor, ecchymosis, petechia, hematuria, or bloody stool.

Forty days prior to admission, the child received external fixation with the steel needles for left femoral neck fracture elsewhere. Pathological biopsy was conducted and a diagnosis of B-cell acute lymphoblastic leukemia/lymphoma (B-ALL/LBL) was made. Twenty days prior to admission, the child suffered from recurrent vomiting, progressive weight loss, bedsore, abnormal renal function, and electrolyte disorder. He was treated elsewhere without improvement.

On admission, the bone marrow smear revealed ALL (type L2).

Initial physical examination revealed: temperature: 37°C, pulse: 106 times/minute, respiration rate: 20 times/minute, blood pressure: 129/94 mmHg, clear consciousness, poor response to stimulation, emaciated appearance. Bilateral inguinal lymph nodes were enlarged with the maximum diameter of around 1 cm, and moderate range of motion. The superficial lymph nodes in both sides of the neck and armpits were not palpable or swollen. The respiratory sound of both lungs was clear without dry or wet rales. The heart sound was strong without pathological murmur. The abdomen was soft, and the liver and spleen were not palpable or swollen. Bedsores were noticed on both sides of the posterior iliac and the outside of the left knee. A longitudinal surgical incision about 10 cm long could be seen at the root of the left thigh. The incision healed well without redness, swelling, or seepage. Four fixed steel needles were noticed on the outside of the left hip, with broken skin and a little exudation on the surface. The remaining skin had no ecchymosis or subcutaneous hemorrhage. The local skin temperature was normal without tenderness or rupture.

#### Laboratory and Imaging Examination

Whole blood test (4 May) revealed: white blood cell: 13.79 × 10^9^/L, absolute neutrophil count: 10.78 × 10^9^/L, hemoglobin: 96 g/L, platelet: 270 × 10^9^/L. Blood electrolytes (7 April) showed: Ca: > 3.49 mmol/L, Cl: 90.1 mmol/L, P: 1.93 mmol/L.

Computed tomography of skull, lumbar spine, femur, and pelvis (23 March) demonstrated scattered nodular osteolytic bone destruction of skull, potential Langerhans histiocytosis, and potential neoplastic lesions. Radiographic examination of 12th thoracic vertebra to 5th lumbar vertebra, bilateral iliac bones, sacral bone, and bilateral thigh bone showed multiple bone destruction of bilateral tibias and fibulas. Some lesions revealed high density shadow and potential neoplastic lesions. Pathological fracture of left femoral neck with swelling and effusion was observed.

Pathological biopsy (27 March) supported the diagnosis of B-cell acute lymphoblastic leukemia/lymphoma (B-ALL/LBL). Immunohistochemistry showed: tumor cell CD79a (+), CD20 (+), TdT (+), CD99 (+), CD10 (+), bcl-6 (+), CD34 (+), c-myc (+, 40–50%), P53 (+, 5–10%), Ki-67 (+, > 80%). Gene rearrangement monitor found lower amplification peaks of IgH and IgK clones.

Bone marrow smear (17 April) revealed acute lymphoblastic leukemia (type L2). ALL common fusion gene test showed negative results. FISH test (12 May) revealed negative results. Karyotype analysis (18 May) showed 47, XY, + 12[3]/46, XY[2].

Thyroid function test (6 May) revealed: T3: 0.57 nmol/L, FT3: 2.0 pmol/L. Renal function test (5 May) showed: urea: 8.1 mmol/L, creatinine: 92 μmol/L, uric acid (7 May): 552 μmol/L. Total parathyroid hormone (6 May) level was 9.5 pg/mL. Vitamin D (6 May) level was 10.5 ng/ml.

**FIGURE 1:**
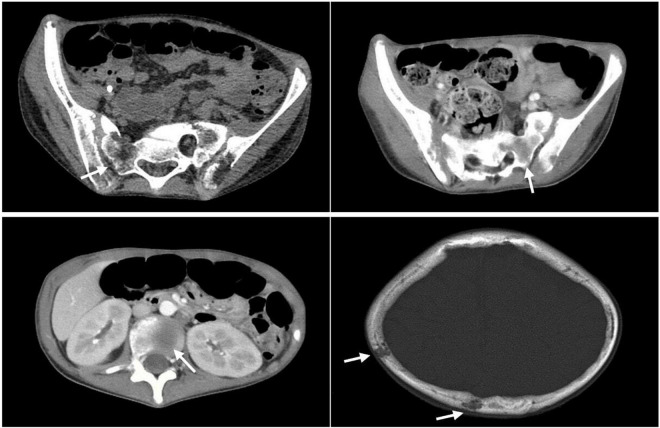
Computed tomography of skull and pelvis showing multiple osteolytic lesions.

Computed tomography (6 May) of skull and pelvis demonstrated multiple osteolytic lesions in skull, bilateral humerus, most vertebrae of thoracolumbar spine, and some accessory bones, sacrum, bilateral iliac bone, sciatic bone, pubis, and bilateral femur (Figure 1). Pelvic X-ray (13 May) showed multiple bone destruction of bilateral iliac bones, sciatic bone, pubic bone, and bilateral thigh bone. In addition, pathological fracture of left femoral neck and internal fixation shadow was found.

#### Treatment and Outcome

**FIGURE 2:**
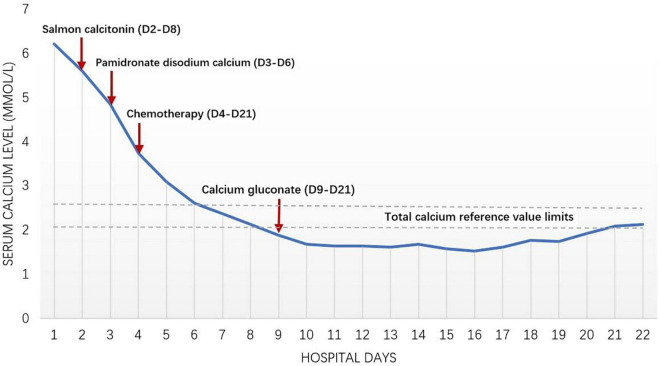
Clinical courses; changes of serum calcium level.

After admission, MICM test confirmed the diagnosis of ALL (type L2, B-cell, negative Fusion Gene, 47, XY, + 12). As the abnormal increase of blood calcium level (>3.49 mmol/L) might be related to bone destruction, salmon calcitonin combined with pamidronate disodium calcium was given. “Numbness” occurred in limbs and *angulus oris* after treatment. Blood calcium level decreased to 1.53 mmol/L, then salmon calcitonin and pamidronate disodium were held (Figure 2). When the blood calcium level and renal function were normalized, the child was treated based on the Chinese Children Cancer Group Acute Lymphoblastic Leukemia (CCCG-ALL-2015) induced remission treatment strategy (dexamethasone, prednisone acetate, vincristine, daunorubicin, and PEG-ASP) on 8 May 2020. Afterward, the D19 bone marrow smear showed complete remission with MRD < 0.01%. The child continued to receive consolidate therapy, and the bone marrow continued to show complete remission.

### Literature Review

A total of 59 articles on children with acute lymphoblastic leukemia complicated with hypercalcemia were retrieved, including 11 in Chinese and 48 in English with 98 children in total. The articles were mainly comprised of case reports (57 reports), and 2 case series. A retrospective study conducted by St. Jude Children’s hospital in the United States between 1962 and 1991 included 25 children with tumor complicated with hypercalcemia. The results revealed that ALL was the most common type of tumor that occurred with hypercalcemia in children (10 cases), with an average age of 11.5 years old (2). Another retrospective study conducted between 1990 and 2005 in Japan included 22 children with ALL complicated with hypercalcemia. Eleven children were older than 10 years old, 14 children had osteolytic lesions, and 8 children had no immature cells in the peripheral hemogram (5). Case reports demonstrated that the peripheral hemogram was normal or slightly changed in most children when ALL was diagnosed. No obvious swelling of liver, spleen, and lymph nodes was observed. No immature cells were detected in the peripheral blood smear. Multiple osteolysis was most commonly found in imaging (6).

## Discussion

The incidence of hypercalcemia in childhood malignant tumors is between 0.4 and 1.3%. It can be seen in Ewing’s sarcoma, neuroblastoma, rhabdomyosarcoma, non-Hodgkin’s lymphoma, neuroblastoma, leukemia, etc. The occurrence of hypercalcemia is related to factors including age > 10 years old, normal or decreased leukocyte count, positive t (17:19), pre-B phenotype combined with the expression of myeloid antigens such as CD13 and CD33. The hemogram of most child patients is completely normal (2, 5, 7, 8). In this case, the disease onset was atypical, mainly presented with non-specific symptoms such as vomiting and bone pain. The peripheral hemogram remained normal without immature cells. It was misleading with symptoms similar to gastrointestinal diseases and surgical diseases. B-ALL/LBL was not suggested in the pathological report of postoperative biopsy until the child had pathological fractures. At that time, the peripheral hemogram of the child was still normal, and the bone pathological biopsy results prompted the clinician to conduct bone marrow smear for diagnosis, which revealed ALL. If the child with bone pain was examined by bone puncture earlier, earlier diagnosis and earlier initiation of treatment might avoid the occurrence of pathological fractures and other related complications.

Malignant tumor is one of the causes of hypercalcemia in children, which mainly involves the following mechanisms: (1) secreting humoral factors such as parathyroid hormone-related protein (PTHrP), tumor necrosis factor (TNF) α and β, transforming growth factor β, Interleukin-1 β, Interleukin-6 and vitamin D sterol, which clinically presented as the increase of serum PTHrP, the decrease of parathyroid hormone (PTH), and the normal or decrease of 1,25-dihydroxyvitamin D (2) osteolytic metastasis with releasing of cytokines locally, which is characterized by decreased PTHrP, PTH, low 1,25-dihydroxyvitamin D or in the normal low limit (3) producing 1,25-dihydroxyvitamin D, which is characterized by an increase in 1,25-dihydroxyvitamin D level (9, 10). In this case, undetectable serum PTHrP level, low PTH (9.5 pg/mL) and low vitamin D (10.5 ng/mL) made it less likely to be hypercalcemia triggered by abnormal secretion of parathyroid hormone. Osteolytic damage caused by infiltration and destruction of bone marrow by leukemia cells was considered instead.

The principles of treating malignancy-induced hypercalcemia (MIH) include reducing blood calcium level and treating the primary disease. Strategies to lower blood calcium include rehydration, diuresis, drug treatment (calcitonin, bisphosphonates, etc.), and dialysis. Calcitonin can increase urinary calcium excretion and interfere with the function of osteoclasts. It takes effect within 4–6 h, and can reduce the blood calcium level by 0.3–0.5 mmol/L at most. The adverse drug reactions are tolerable. Bisphosphonates can interfere with osteoclasts and inhibit bone reabsorption. Adverse reactions are mainly influenza-like symptoms (fever, joint pain, myalgia, fatigue, and bone pain), hypocalcemia and hypophosphatemia. The American Society of Clinical Oncology (ASCO) and the food and Drug Administration (FDA) recommend zoledronic acid and pamidronate disodium for the treatment of adult tumor-related hypercalcemia (11). Evidence for their use in children is limited. Kolyva et al. reported a case of a 11.5-year-old child with ALL combined hypercalcemia. The blood calcium decreased to normal range within 48 h with one dose of zoledronic acid (0.05 mg/kg) (6). Bota et al. reported that a 6-year-old child with ALL combined with hypercalcemia developed hypocalcemia 2 days after receiving calcitonin (4 U/kg, q12 h) and zoledronic acid (2 mg) (12). Therefore, in order to avoid rebound hypocalcemia, it is recommended to initiate zoledronic acid (0.0125–0.05 mg/kg) and pamidronate disodium (0.25–2 mg/kg, maximum 60 mg) at a small dose, and the duration of therapy should be limited to avoid hypocalcemia, hand and foot convulsions, etc. (13). The combination of calcitonin and bisphosphonates can reduce blood calcium faster (14). If there is no response to drug therapy or the clinical condition is critical, dialysis may be adopted. Patients with refractory hypercalcemia or severe renal dysfunction can choose dinozumab (9). In this case, the child was recovering smoothly with chemotherapy and the renal function was normal. Initial blood calcium level was > 3.49 mmol/L. One day of rehydration, furosemide for diuresis, and calcitonin (4–8IU/kg*D) brought poor response. With the combination of disodium pamidronate (1 mg/kg*D), the blood calcium decreased to normal range with 3-day treatments. Transient hypocalcemia characterized by numbness in hands, feet, and lips occurred during continuous use. Therefore, in the course of calcitonin and pamidronate disodium treatment, it is necessary to closely monitor the blood calcium changes to avoid over correction and hypocalcemia.

## Conclusion

Hypercalcemia alone or combined with osteolytic lesions may be the main clinical presentation of ALL in children. Although the case is rare, clinicians should be extremely vigilant, especially in the early stage of disease onset when the peripheral hemogram is completely normal, and no typical clinical presentations of ALL are noticed. It is very likely to be misdiagnosed or mistreated. The bone marrow examination should be arranged in time. In addition, hypercalcemia caused by ALL usually responded poorly to hydration and diuretics. Drug treatment lowering blood calcium, such as salmon calcitonin combined with pamidronate disodium, should be initiated in time. At the same time, blood calcium changes should be monitored closely to avoid therapeutic hypocalcemia.

## Data Availability Statement

The original contributions presented in this study are included in the article/Supplementary Material, further inquiries can be directed to the corresponding author/s.

## Ethics Statement

The studies involving human participants were reviewed and approved by West China Second University Hospital Ethical Committee. Written informed consent to participate in this study was provided by the participants’ legal guardian/next of kin.

## Author Contributions

MC and XL: data collection. XL and JN: data analysis. MC and JN: data interpretation. JN, MC, and XL: manuscript drafting. All authors contributed to the article and approved the submitted version.

## Conflict of Interest

The authors declare that the research was conducted in the absence of any commercial or financial relationships that could be construed as a potential conflict of interest.

## Publisher’s Note

All claims expressed in this article are solely those of the authors and do not necessarily represent those of their affiliated organizations, or those of the publisher, the editors and the reviewers. Any product that may be evaluated in this article, or claim that may be made by its manufacturer, is not guaranteed or endorsed by the publisher.
