# Supplementary material for: Hypercalcemia and Osteolytic Lesions as Presenting Symptoms of Acute Lymphoblastic Leukemia in Children: Case Report and Literature Review
Source: Front Pediatr. 2022 Jun 9;10:923297. doi: 10.3389/fped.2022.923297 (PMC9218481; doi:10.3389/fped.2022.923297)
Supplement: Supplementary file 1 [file Data_Sheet_1.pdf]

Supplementary Figures

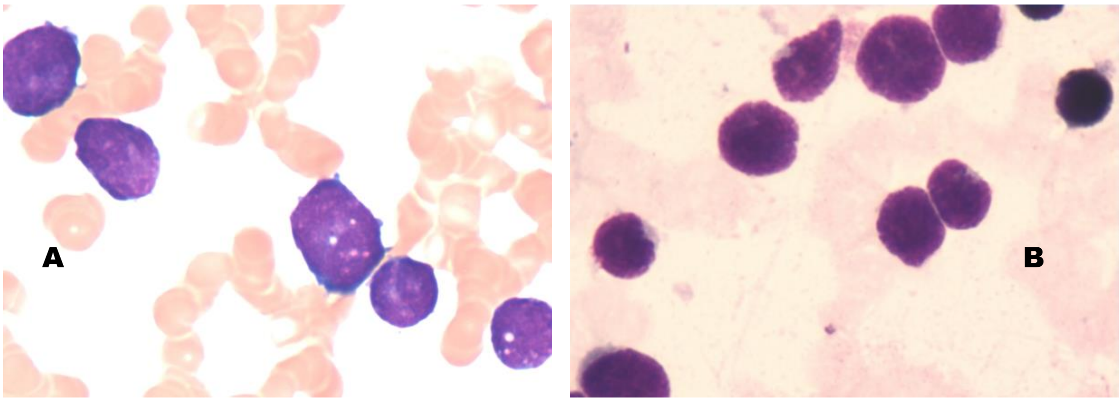

Figure 1. A. Giemsa stain of bone marrow cells (X1,000) B. Peroxidase stain of bone marrow cells (X1,000)

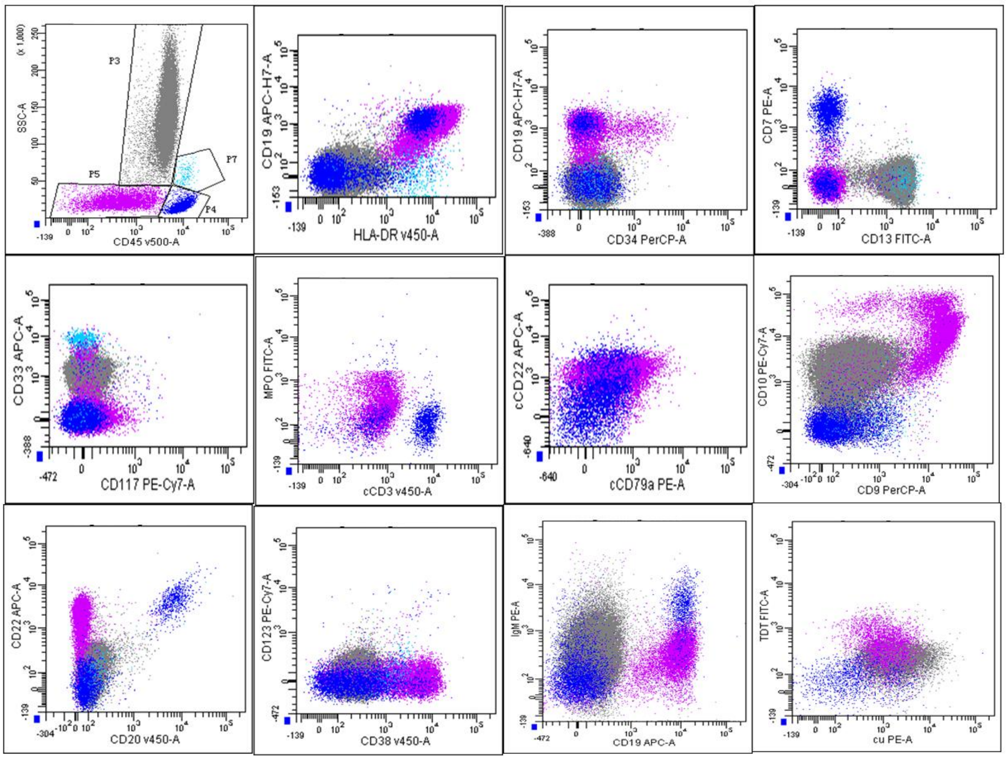

Figure 2. Flow cytometric analysis of bone marrow cells

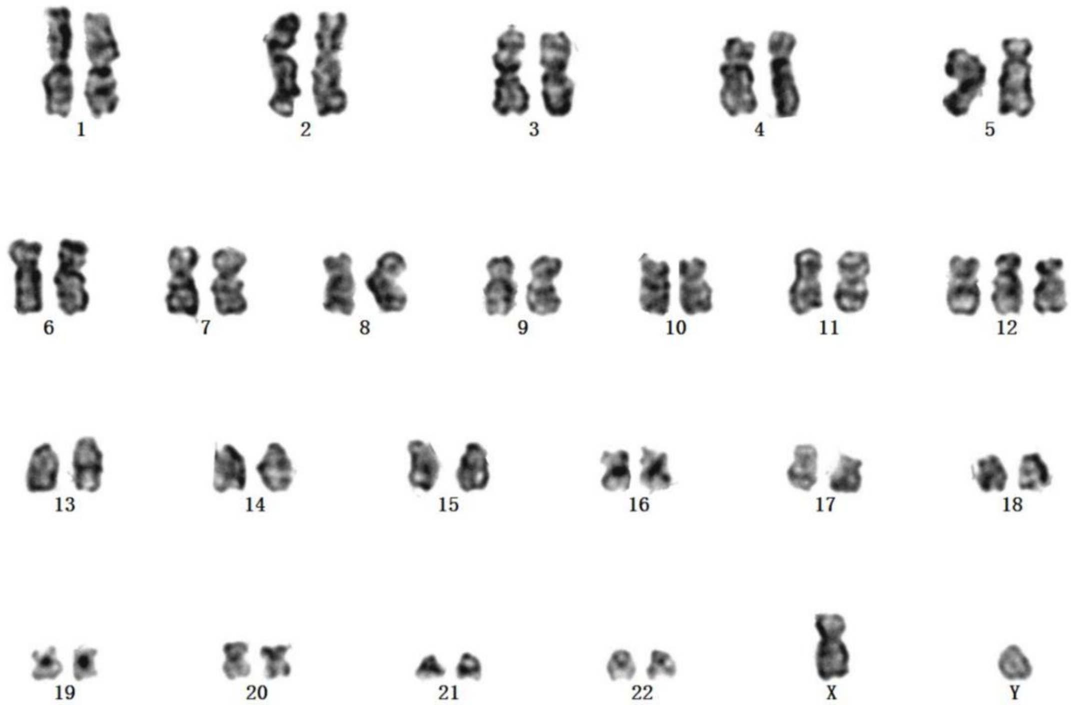

Figure 3. Cytogenetic analysis of bone marrow cells

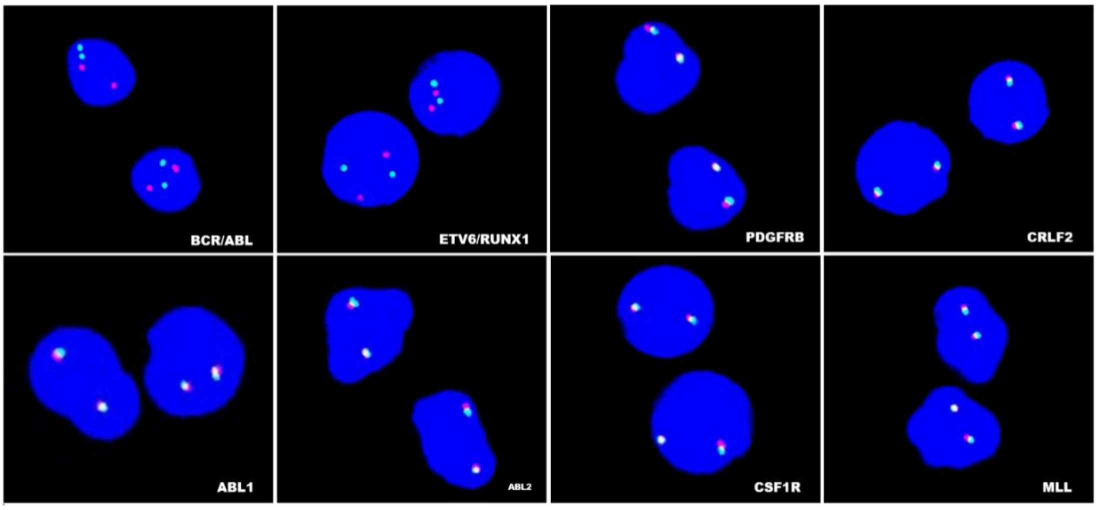

Figure 4. FISH testing of bone marrow cells
